# Influence of Educational Films on Antiviral Prescription for COVID-19: Insights from Web-Based Survey in Japan

**DOI:** 10.3390/antibiotics14030276

**Published:** 2025-03-07

**Authors:** Kosaku Komiya, Akihiko Hagiwara, Yuichiro Shindo, Kazufumi Takamatsu, Naoki Nishimura, Yukako Takechi, Eiki Ichihara, Takahiro Takazono, Shinyu Izumi, Shimpei Gotoh, Seiichiro Sakao, Takehiro Izumo, Kazuko Yamamoto, Kazuhiro Yatera, Hiroshi Kakeya, Yoko Shibata, Keisuke Tomii, Hironori Sagara, Yuka Sasaki, Toyohiro Hirai, Akihito Yokoyama, Hiroshi Mukae, Takashi Ogura

**Affiliations:** 1 Respiratory Medicine and Infectious Diseases, Oita University Faculty of Medicine, 1-1 Idaigaoka, Hasama-machi, Yufu 879-5593, Japan; a-hagiwara@oita-u.ac.jp; 2 Department of Respiratory Medicine, Nagoya University Graduate School of Medicine, 65 Tsurumai-cho, Showa-ku, Nagoya 466-8550, Japan; 3 Department of Respiratory Medicine and Allergology, Kochi Medical School, Kochi University, Kohasu, Oko-cho, Nankoku 783-8505, Japan; 4 Department of Respiratory Medicine, National Center for Global Health and Medicine, 1-21-1, Toyama, Shinjuku-ku, Tokyo 162-8655, Japan; 5 Iki-iki Clinic, 2-34 Minamisaiwai-cho, Saiwai-ku, Kawasaki 212-0016, Japan; 6 Center for Clinical Oncology, Okayama University Hospital, 2-5-1 Shikata-cho, Kita-ku, Okayama 700-8558, Japan; 7 Department of Respiratory Medicine, Nagasaki University Graduate School of Biomedical Sciences, 1-7-1 Sakamoto, Nagasaki 852-8501, Japan; 8 Department of Clinical Application, Center for iPS Cell, Research and Application, Kyoto University, 53 Shogoin Kawahara-cho, Sakyo-ku, Kyoto 606-8507, Japan; 9 Department of Pulmonary Medicine, School of Medicine, International University of Health and Welfare, 4-3 Kozunomori, Narita 286-8686, Japan; 10 Department of Respiratory Medicine, Japanese Red Cross Medical Center, 4-1-22 Hiroo, Shibuya-ku, Tokyo 150-8935, Japan; 11 Division of Infectious, Respiratory, and Digestive Medicine, First Department of Internal Medicine, University of the Ryukyus Graduate School of Medicine, Kiyuna 1076, Ginowan 901-2720, Japan; 12 Department of Respiratory Medicine, School of Medicine, University of Occupational and Environmental Health, Japan, 1-1 Iseigaoka, Yahatanishi-ku, Kitakyushu 807-8555, Japan; 13 Department of Infection Control Science, Osaka Metropolitan University Graduate School of Medicine, 1-4-3, Asahi-machi, Abeno-ku, Osaka 545-8585, Japan; 14 Department of Pulmonary Medicine, Fukushima Medical University School of Medicine, 1 Hikarigaoka, Fukushima 960-1295, Japan; 15 Department of Respiratory Medicine, Kobe City Medical Center General Hospital, 2-1-1 Minatojimaminamimachi, Chuo-ku, Kobe 650-0047, Japan; 16 Department of Medicine, Division of Respiratory Medicine and Allergology, Showa University School of Medicine, 1-5-8, Hatanodai, Shinagawa-ku, Tokyo 142-8666, Japan; 17 Center for Pulmonary Disease, National Hospital Organization Tokyo Hospital, 3-1-1, Takeoka, Kiyose-shi 204-8585, Japan; 18 Department of Respiratory Medicine, Graduate School of Medicine, Kyoto University, 54 Shogo-in Kawahara-cho, Sakyo-ku, Kyoto 606-8507, Japan; 19 Department of Respiratory Medicine, Kanagawa Cardiovascular and Respiratory Center, 6-16-1 Tomiokahigashi, Kanazawa-ku, Yokohama 236-0051, Japan

**Keywords:** antiviral, COVID-19, prescription, education

## Abstract

**Background:** Prescribing antiviral agents for severe acute respiratory syndrome coronavirus 2 requires careful consideration based on the patient’s risk factors for severe disease progression and their vaccination status. However, effective interventions ensuring the appropriate use of antiviral agents by physicians have yet to be fully established. Thus, this study evaluated the impact of an educational film on antiviral prescription rates for coronavirus disease 2019 (COVID-19). **Methods:** This prospective, nationwide, web-based survey enrolled 1500 physicians. They were instructed to view a short educational film and assess the necessity of prescribing antiviral agents in 16 fictitious scenarios featuring adult patients with COVID-19 with varying risk factors for severe disease and vaccination statuses. We compared the antiviral prescription rates before and after viewing the educational film. **Results:** There was a significant increase in the antiviral prescription rates after viewing the educational film, particularly nirmatrelvir/ritonavir prescribed in cases involving immunocompromised patients (from 31.3% to 49.4%) and those with obesity (from 15.1% to 33.7%) who were unvaccinated and had no risk of drug interactions. However, viewing the educational film made little to no impact on the prescription rates for the patients with hypertension and hyperlipidemia or those with no underlying conditions. **Conclusions:** Short educational films may promote the appropriate use of antiviral agents for COVID-19. However, their impact on altering prescription behavior appears limited and varies according to the clinical context.

## 1. Introduction

The coronavirus disease 2019 (COVID-19) pandemic was mitigated by widespread social immunization achieved via vaccination and natural infection. However, individuals with risk factors for severe disease progression, such as those with immunocompromised conditions, morbid obesity, or severe renal dysfunction, remain at risk of requiring intensive care. These high-risk populations are particularly vulnerable, necessitating prompt antiviral treatment upon COVID-19 diagnosis [1]. However, the efficacy of antiviral drugs in preventing disease progression has been reported to diminish in individuals who maintain immunity against severe acute respiratory syndrome coronavirus 2 (SARS-CoV-2) through additional booster vaccinations. For example, the treatment of symptomatic COVID-19 with nirmatrelvir/ritonavir showed a relative risk reduction (89%) in hospitalization or death by day 28 among nonhospitalized high-risk patients [2]. In contrast, it was recently reported that nirmatrelvir/ritonavir does not significantly impact the time to sustained symptom alleviation or reduce the risk of hospitalization or death in patients at standard risk or in those fully vaccinated who had at least one risk factor for severe disease [3]. Similarly, while molnupiravir initially showed a relative risk reduction (30%) in hospitalization or death among at-risk, unvaccinated adults in 2020 [4], the subsequent evidence did not reveal a significant benefit in reducing COVID-19-associated hospitalizations or deaths among high-risk vaccinated individuals in the community [5].

In Japan, the proportion of individuals receiving COVID-19 vaccine boosters has declined as daily life returned to normal, while some countries have decided to maintain specific vaccination levels for high-risk populations [6,7]. Given the limited duration of vaccine-induced immunity, the number of individuals at risk for severe COVID-19 progression who have not received additional booster doses is likely increasing. Thus, to prevent a resurgence of severe COVID-19 cases, physicians must recognize the risk factors for severe disease progression in the context of patient’s current vaccination status. However, there is a lack of surveys assessing physicians’ knowledge of the risk factors for severe COVID-19, particularly when taking into account vaccination status. Additionally, there is a paucity of interventional studies evaluating the impact of educational tools on antiviral agent prescription behaviors. To address these gaps, we performed a nationwide survey assessing the antiviral prescription patterns among physicians managing COVID-19 patients before and after watching an educational film with 16 fictitious scenarios of patients with mild COVID-19 who had various risk factors for severe disease and different vaccination statuses.

## 2. Results

### 2.1. Participants’ Backgrounds

We enrolled 1500 physicians (996 generalists, 228 pulmonologists, 136 otolaryngologists, 15 gastroenterologists, 12 pediatricians, 12 psychiatrists, 11 cardiologists, 10 orthopedic surgeons, 10 neurologists, 10 gastrointestinal surgeons, and 60 physicians from other specialties), among whom 160 (10.7%) were female. The participants’ age distribution was as follows: 81 (5.4%) aged ≥70 years, 469 (31.3%) in their 60s, 420 (28.0%) in their 50s, 298 (19.9%) in their 40s, 196 (13.1%) in their 30s, and 36 (2.4%) in their 20s. Regarding the workplace settings, 891 (59.4%) physicians were employed in hospitals, and 609 (40.6%) in clinics (Table 1).

### 2.2. Rates of Antiviral Prescription Before Viewing the Educational Film

**Figure 1:**
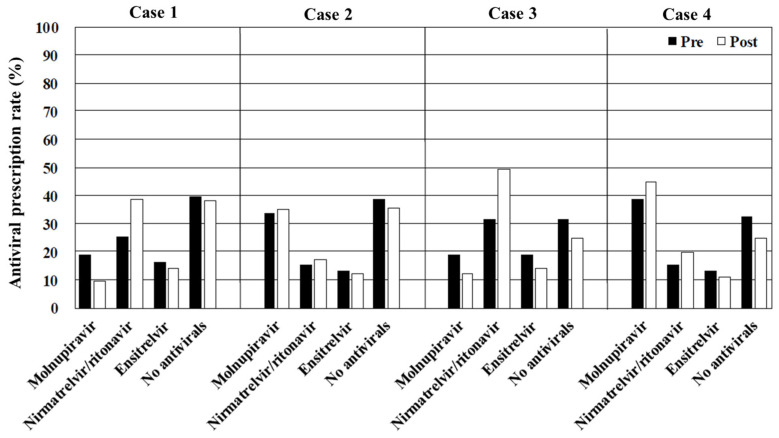
The overall rates of antiviral prescription in cases 1–4. Pre, before viewing the educational film; post, after viewing the educational film.

**Figure 2:**
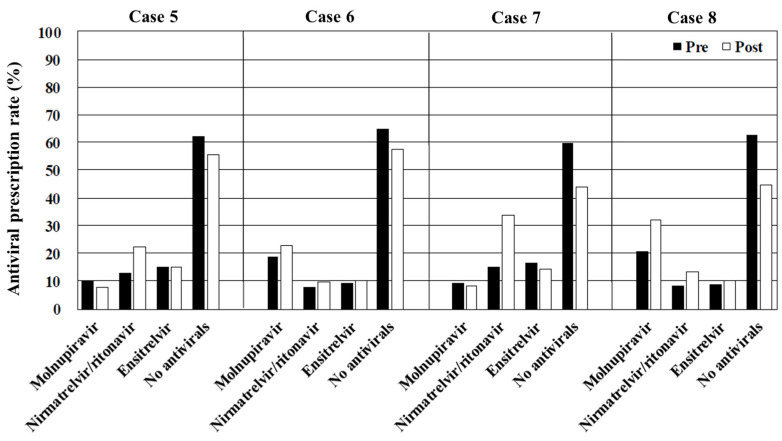
The overall rates of antiviral prescription in cases 5–8. Pre, before viewing the educational film; post, after viewing the educational film.

**Figure 3:**
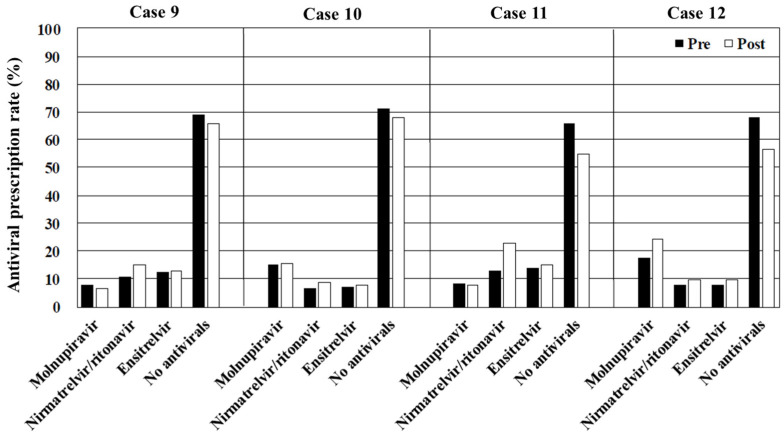
The overall rates of antiviral prescription in cases 9–12. Pre, before viewing the educational film; post, after viewing the educational film.

**Figure 4:**
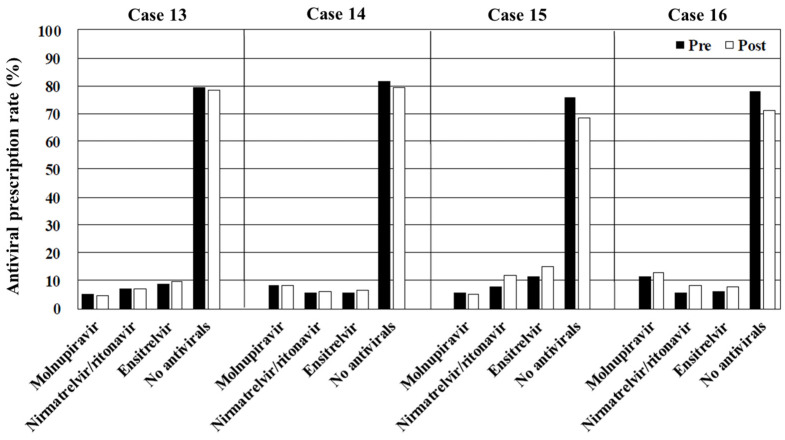
The overall rates of antiviral prescription in cases 13–16. Pre, before viewing the educational film; post, after viewing the educational film.

The overall rates of antiviral prescription, irrespective of the drug type, decreased as the risk of disease progression diminished by >60% in cases 1–4 (immunosuppressed) and approximately 40% in cases 5–8 (obesity), 30% in cases 9–12 (hypertension or hyperlipidemia), and 20% in cases 13–16 (no risk of disease progression). Molnupiravir was the most frequently prescribed antiviral in cases where interactions with CYP3A-metabolized drugs contraindicated the use of nirmatrelvir/ritonavir and ensitrelvir (cases 2, 4, 6, 8, 10, 12, 14, and 16). In contrast, the primary choice in case 1 (immunosuppressed, vaccinated, and no CYP3A-metabolized drugs) and case 3 (immunosuppressed, unvaccinated, and no CYP3A-metabolized drugs) was nirmatrelvir/ritonavir, and ensitrelvir was preferred in cases 5, 7, 9, 11, 13, and 15 (immunocompetent and no CYP3A-metabolized drugs) (Figure 1, Figure 2, Figure 3 and Figure 4). Table 2, Table 3, Table 4 and Table 5 present the top three reasons for prescribing or withholding antiviral therapy for each group with underlying conditions (i.e., immunosuppression, obesity, hypertension or hyperlipidemia, and no risk factors for disease progression). Regardless of the patients’ backgrounds, the presence of mild symptoms was the most common reason for withholding antiviral treatment. The primary factors influencing the decision to prescribe antivirals included the perceived clinical benefit, the physician’s prior experience, and concerns regarding drug interactions.

### 2.3. Effects of the Educational Film on the Appropriate Use of Antivirals

Viewing the educational film significantly influenced the participants’ treatment decisions regarding COVID-19 in cases 1, 3, 4, 5, 6, 7, 8, 9, 11, 12, 15, and 16 (*p* < 0.001, <0.001, <0.001, <0.001, =0.001, <0.001, <0.001, =0.009, <0.001, <0.001, <0.001, and <0.001, respectively). In contrast, there were no significant differences in cases 2, 10, 13, and 14 (*p* = 0.157, 0.141, 0.612, and 0.552, respectively). The overall rate of antiviral prescription increased across all the cases after viewing the film. Notable increases were observed in the prescription of nirmatrelvir/ritonavir, which rose from 25.5% to 38.3% in case 1 (immunosuppressed, vaccinated, and no CYP3A-metabolized drugs), 31.3% to 49.4% in case 3 (immunosuppressed, unvaccinated, and no CYP3A-metabolized drugs), 12.8% to 22.3% in case 5 (obesity, vaccinated, and no CYP3A-metabolized drugs), and 15.1% to 33.7% in case 7 (obesity, unvaccinated, and no CYP3A-metabolized drugs). There was also an apparent increase in molnupiravir prescription in the cases involving patients taking CYP3A-metabolized drugs. The proportion of physicians citing the physician’s prior experience as a reason for prescribing antivirals decreased across all the cases after viewing the film. Subgroup analyses comparing the generalists and the specialists did not reveal specific trends or significant differences between the two groups (Supplementary Figures S1–S8).

## 3. Discussion

We demonstrated that viewing an educational film of 16 fictitious scenarios of adult patients with COVID-19 with varying risk factors for severe disease and different vaccination statuses significantly increased the rate of antiviral prescription, particularly nirmatrelvir/ritonavir, among the patients who were immunosuppressed or had obesity. Despite this improvement, approximately 20–40% of the participating physicians indicated that they would not prescribe antivirals, even for immunosuppressed patients where such treatment was necessary to prevent severe disease progression. Molnupiravir emerged as the most frequently chosen antiviral agent for the patients taking CYP3A-metabolized medications. Furthermore, the educational film effectively reduced the proportion of physicians who relied solely on their personal experience with antiviral medications when making prescription decisions, promoting a more evidence-based approach.

In cases 1–4 (immunosuppressed), the prescription rate for nirmatrelvir/ritonavir was highest among the patients not taking CYP3A-metabolized medications, while molnupiravir was most frequently prescribed for those who were. Notably, approximately 30% of the physicians stated that they would not prescribe antivirals in these scenarios. Underlying conditions, such as immunosuppression caused by corticosteroid or other immunosuppressive medications and solid organ or blood stem cell transplantation, are well-established risk factors for severe disease progression [8,9,10]. Furthermore, immunocompromised patients show more prolonged COVID-19 shedding compared with that of immunocompetent individuals [11]. Given the inadequate antibody responses to vaccination in this population, antiviral therapy is strongly recommended, regardless of their COVID-19 vaccination status [12,13]. Although the educational film emphasized the importance of antiviral treatment for immunosuppressed patients, the increase in the prescription rate before and after viewing the film remained insufficient. This may be because the educational content was primarily based on the results of randomized controlled trials (RCTs) instead of real-world clinical case presentations of COVID-19. The most frequently cited reason for not prescribing antivirals was the perception of mild symptoms, suggesting that physicians underestimate the heightened risk of disease progression in immunosuppressed patients. Thus, it is crucial to recognize that the decision to administer oral antivirals to patients with mild COVID-19 must be based on the risk of disease progression. This critical point was inadequately emphasized in the educational film, potentially contributing to the suboptimal impact on antiviral prescription practices.

The prescription patterns in cases 5–8 (obesity) were similar to those in the scenarios involving patients with immunosuppression (cases 1–4), with an overall antiviral prescription rate of approximately 50%. Other underlying conditions, including obesity, dialysis, diabetes, and cancer, are also recognized as significant risk factors for severe disease progression [10,14,15]. Furthermore, patients with severe COVID-19 tend to have a higher body mass index compared with those with nonsevere disease, underscoring the necessity for antiviral therapy in cases of obesity [16,17]. Obesity is associated with poor outcomes in patients with COVID-19 due to multiple mechanisms, such as chronic inflammation driven by increased cytokine production, impaired respiratory function, pulmonary perfusion issues (e.g., vascular thrombosis), and other vascular complications [18,19]. Although immunosuppression diminishes vaccine effectiveness [12], there is no evidence suggesting that obesity directly weakens vaccine efficacy. Nevertheless, vaccine effectiveness declines over time. Individuals aged 18–64 years who received at least three doses showed decreased vaccine effectiveness (46.4% at 3–5 months vs. 18.3% at 12–14 months post-vaccination), as did those aged ≥65 years, (65.3% at 3–5 months vs. 52.3% at 12–14 months) [20]. Notably, meta-analysis revealed that hybrid immunity (i.e., prior SARS-CoV-2 infection and vaccination) provided 97.4% effectiveness against hospitalization and disease progression at 12 months [21]. Antivirals are strongly recommended for patients with obesity who do not have a recent vaccination history. However, the educational film’s impact on prescribing practices in this population was suboptimal, increasing from 40.5% to 56.1% in case 7 (obesity, unvaccinated, and no CYP3A-metabolized drugs) and from 37.0% to 55.5% in case 8 (obesity, unvaccinated, and CYP3A-metabolized drugs). Symptomatic treatment may be considered a viable option for patients with a recent vaccination history.

In cases 9–16 (hypertension or hyperlipidemia and no risk of severe disease progression), the antiviral prescription rates were relatively lower compared with those for the patients with an immunosuppressed status or obesity. Chronic conditions, such as hypertension and hyperlipidemia alone, are not classified as high-risk factors for severe disease progression [10]. Following the viewing of the educational film, there was a modest increase in the prescription rates for nirmatrelvir/ritonavir, from 10.9% to 14.8% in case 9 (hypertension or hyperlipidemia, vaccinated, and no CYP3A-metabolized drugs) and 12.7% to 22.5% in case 11 (hypertension or hyperlipidemia, unvaccinated, and no CYP3A-metabolized drugs). However, symptomatic treatment or ensitrelvir (an antiviral that shortens symptom duration) may be more appropriate in such cases [22]. In fact, ensitrelvir was the most frequently selected antiviral for the patients without a risk of disease progression and not taking CYP3A-metabolized medication, both before and after viewing the educational film. This preference may be attributed to clinical trials, where ensitrelvir has mainly been tested on patients without risk factors for severe disease progression [22].

Overall, our study demonstrated increased antiviral prescription rates after viewing the educational film, particularly among the patients at higher risk for disease progression. Thus, the film appeared to exert a positive influence on appropriate antiviral use. However, the rate of antiviral prescriptions for immunosuppressed patients was lower than expected, highlighting an area for improvement. Notably, the proportion of physicians citing a physician’s prior experience as the reason for their prescription decisions decreased, suggesting a shift toward evidence-based practices. Thus, educational interventions using short films may play a valuable role in promoting such practices. To enhance the effectiveness of these interventions, future educational films should incorporate case presentations alongside scientific evidence from RCTs. This combination may enhance physicians’ engagement and improve their ability to apply evidence-based approaches in real-world clinical scenarios.

The strength of this study lies in its distinction as, to our knowledge, the first large-scale investigation of the effects of educational interventions on the appropriate use of antivirals for COVID-19. However, there were several limitations. First, the film presented fictitious scenarios with limited information, including the patients’ underlying conditions, vaccination status, concomitant medications, chief complaints, and chest X-ray findings, to minimize the burden on the participants. Thus, the findings may not be a true reflection of real-world clinical practice. Second, the cost of drugs may influence a physician’s decision to prescribe antivirals in clinical settings. In Japan, the approximate costs per treatment course of molnupiravir, nirmatrelvir/ritonavir, and ensitrelvir are USD 150, 200, and 100, respectively, for patients with a standard income. Third, the educational film emphasized the prevention of severe disease progression rather than a symptomatic cure. Consequently, the results do not reflect the impact of antiviral agents on COVID-19 symptoms. Last, because this study assessed the physicians’ intentions to prescribe antivirals immediately after viewing the educational film, the long-term effects of the intervention are unclear.

In conclusion, the educational film effectively increased the overall antiviral prescription rates. However, subgroup analysis revealed that the prescribing rates remained suboptimal among the high-risk patients, particularly those who were immunosuppressed. This disparity underscores the need for targeted interventions to ensure appropriate antiviral use in vulnerable populations. Enhancing educational programs by incorporating the clinical experiences of severe COVID-19 cases and strengthening communication between high-level medical institutions and clinics may further improve prescription practices. This approach is particularly crucial for clinic physicians, who may rarely encounter severe COVID-19 cases, but play a key role in early intervention for high-risk patients.

## 4. Materials and Methods

### 4.1. Study Design and Participants

This prospective, web-based interventional study was conducted using an online network platform (PLAMED Inc., Tokyo, Japan) that facilitates medical care surveys for its physician members. We targeted 1500 physicians registered on the platform and collected the background data, including age, sex, specialty, and workplace. This investigation was part of a campaign promoting the proper use of antivirals for COVID-19 treatment that was organized by the COVID-19 Clinical Expert Opinion Working Group of the Japanese Respiratory Society (JRS).

### 4.2. Fictitious Scenarios

The physicians were asked to determine whether they would prescribe antivirals (molnupiravir, nirmatrelvir/ritonavir, and ensitrelvir) in 16 fictitious scenarios of mild COVID-19 incorporating patients with varied backgrounds, including those with underlying conditions (immunosuppressed status, obesity, hypertension or hyperlipidemia, and no risk factors for disease progression), having had a vaccination within 1 year, and who take CYP3A-metabolized medications (Table 6). They selected reasons for their decisions from multiple options (multiple selections were allowed). The same clinical presentation was used for all scenarios as follows: A patient in his 40s visited the hospital with a persistent fever for 2 days. Chest X-ray revealed no abnormal lung involvement. Following a positive antigen test, mild COVID-19 without hypoxia was diagnosed.

### 4.3. Educational Film

The educational film was developed by members of the JRS committee, and we provided a thorough review of evidence regarding the use of antiviral drugs for COVID-19 treatment, emphasizing the findings from RCTs. The film underscored the criticality of patient characteristics, including the risk of severe disease progression and vaccination status, when interpreting the clinical trial data. Four antiviral drugs are currently approved for use in Japan. This investigation focused on oral treatments suitable for outpatient settings, namely nirmatrelvir/ritonavir, molnupiravir, and ensitrelvir. The key messages of the film were as follows: (1) nirmatrelvir/ritonavir demonstrated an 89% reduction in hospitalization or death among unvaccinated high-risk patients [2], but did not significantly improve symptoms in vaccinated individuals [3]; (2) molnupiravir achieved a 30% reduction in severe outcomes in unvaccinated high-risk patients [4], but no efficacy was evident in vaccinated populations [5]; and (3) ensitrelvir shortened symptom recovery time by approximately 1 day in vaccinated patients, regardless of the risk factors. Notably, an Asian RCT conducted in Japan, Vietnam, and South Korea revealed that ensitrelvir reduced the time to resolution of five persistent COVID-19 symptoms (i.e., stuffy or runny nose, sore throat, cough, fever, and fatigue) from 8 to 7 days, irrespective of the risk factors [22]. The fundamental characteristics of these antiviral agents are summarized in Table 7.

**Figure 5:**
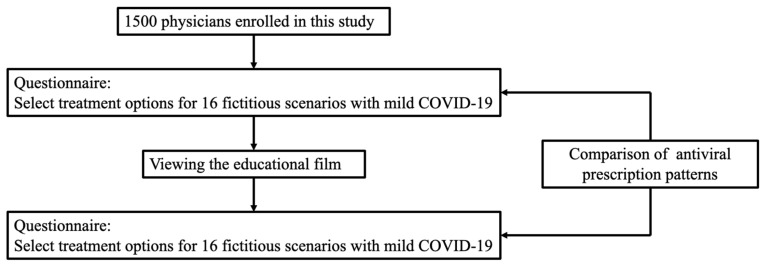
Scheme of this investigational survey.

The film highlighted the major risk factors for severe disease progression, such as advanced age, immunosuppression, severe obesity, and dialysis dependency, emphasizing that although vaccines provide robust protection against severe outcomes, antiviral treatments remain critical for individuals with diminished vaccine efficacy due to these vulnerabilities. The film presented evidence supporting symptomatic management or the use of ensitrelvir for patients without risk factors and the use of nirmatrelvir/ritonavir for high-risk individuals, particularly those who had not received additional booster vaccines. However, because ensitrelvir and nirmatrelvir/ritonavir interact with CYP3A-metabolized drugs, the careful consideration of concomitant medications was deemed crucial. In cases where drug interactions contraindicate the use of nirmatrelvir/ritonavir, molnupiravir or remdesivir were recommended as viable alternatives. The film also recommended that a comprehensive assessment of disease progression risk, vaccination history, and potential drug interactions be used to guide the selection of antiviral medication. The short film (8 min and 29 s) is available in English (https://kaihatsu-test.com/efaddtto-covid19-2024/20241210_eng_1.html) (accessed on 26 January 2025) and Japanese (https://kaihatsu-test.com/efaddtto-covid19-2024/20241210_jpn_1.html) (accessed on 26 January 2025). After watching the film, the participants were asked to decide whether they would prescribe antivirals (molnupiravir, nirmatrelvir/ritonavir, or ensitrelvir) in the same 16 hypothetical scenarios involving patients with mild COVID-19 (Figure 5).

### 4.4. Statistical Analysis

All statistical analyses were performed using SPSS v22 (IBM Japan, Tokyo, Japan). The rates of antiviral prescription before and after viewing the educational film were compared using the chi-square test. Subgroup analyses were also performed following stratification by medical specialty (generalists vs. nongeneralists). *p*-value < 0.05 was considered statistically significant.

## Figures and Tables

**Table 1:** The demographic and professional characteristics of the physicians participating in this study (N = 1500).

Variables	Number (%)
Age	
20s	36 (2.4)
30s	196 (13.1)
40s	298 (19.9)
50s	420 (28.0)
60s	469 (31.3)
70s or older	81 (5.4)
Sex, female	160 (10.7)
Specialty	
Generalists	996 (66.4)
Pulmonologists	228 (15.2)
Otolaryngologists	136 (9.1)
Others	140 (9.3)
Workplace	
Hospital	891 (59.4)
Clinic	609 (40.6)

**Table 2:** Reasons for prescribing or withholding antivirals among immunosuppressed patients before and after viewing educational film.

Case 1							
No Antivirals				Antivirals			
Pre (n = 596)		Post (n = 569)		Pre (n = 904)		Post (n = 931)	
Mild symptoms	548 (91.9)	Mild symptoms	451 (79.3)	Perceived clinical benefit	438 (48.5)	Perceived clinical benefit	662 (71.1)
Vaccination within the past year	200 (33.6)	Vaccination within the past year	173 (30.4)	Physician’s prior experience	323 (35.7)	Physician’s prior experience	160 (17.2)
Concerns about drug cost	121 (20.3)	Concerns about drug cost	69 (12.1)	Potential adverse effects	190 (21.0)	Potential adverse effects	102 (11.0)
Case 2							
No antivirals				Antivirals			
Pre (n = 580)		Post (n = 532)		Pre (n = 920)		Post (n = 968)	
Mild symptoms	482 (83.1)	Mild symptoms	380 (71.4)	Concerns regarding drug interactions	354 (38.5)	Concerns regarding drug interactions	400 (41.3)
Vaccination within the past year	157 (27.1)	Vaccination within the past year	173 (32.5)	Physician’s prior experience	285 (31.0)	Perceived clinical benefit	375 (38.7)
Concerns regarding drug interactions/concerns about drug cost	92 (15.9)	Concerns about drug cost	66 (12.4)	Perceived clinical benefit	272 (29.6)	Physician’s prior experience	160 (16.5)
Case 3							
No antivirals				Antivirals			
Pre (n = 472)		Post (n = 368)		Pre (n = 1028)		Post (n = 1132)	
Mild symptoms	398 (84.3)	Mild symptoms	299 (81.3)	Perceived clinical benefit	517 (50.3)	Perceived clinical benefit	798 (70.5)
Concerns about drug cost	77 (16.3)	Concerns about drug cost	61 (16.6)	Physician’s prior experience	365 (35.5)	Physician’s prior experience	206 (18.2)
No perceived clinical benefit	41 (8.7)	No perceived clinical benefit	24 (6.5)	Potential adverse effects	171 (16.6)	Potential adverse effects	131 (11.6)
Case 4							
No antivirals				Antivirals			
Pre (n = 490)		Post (n = 368)		Pre (n = 1010)		Post (n = 1132)	
Mild symptoms	387 (79.0)	Mild symptoms	285 (77.4)	Concerns regarding drug interactions	395 (39.1)	Concerns regarding drug interactions	491 (43.4)
Concerns regarding drug interactions	68 (13.9)	Concerns about drug cost	57 (15.5)	Physician’s prior experience	296 (29.3)	Perceived clinical benefit	421 (37.2)
Concerns about drug cost	64 (13.1)	Concerns regarding drug interactions	40 (10.9)	Perceived clinical benefit	287 (28.4)	Physician’s prior experience	174 (15.4)

The data are presented as numbers (%). Pre, before viewing the educational film; post, after viewing the educational film.

**Table 3:** Reasons for prescribing or withholding antivirals among patients with obesity before and after viewing educational film.

Case 5							
No Antivirals				Antivirals			
Pre (n = 935)		Post (n = 830)		Pre (n = 565)		Post (n = 670)	
Mild symptoms	834 (89.2)	Mild symptoms	675 (81.3)	Physician’s prior experience	206 (36.5)	Perceived clinical benefit	398 (59.4)
Vaccination within the past year	203 (21.7)	Vaccination within the past year	263 (31.7)	Perceived clinical benefit	202 (35.8)	Physician’s prior experience	113 (16.9)
Concerns about drug cost	122 (13.0)	Concerns about drug cost	109 (13.1)	Concerns about drug cost	123 (21.8)	Concerns about drug cost	112 (16.7)
Case 6							
No antivirals				Antivirals			
Pre (n = 972)		Post (n = 861)		Pre (n = 528)		Post (n = 639)	
Mild symptoms	855 (80.0)	Mild symptoms	682 (79.2)	Concerns regarding drug interactions	185 (35.0)	Concerns regarding drug interactions	254 (39.7)
Vaccination within the past year	246 (25.3)	Vaccination within the past year	295 (34.3)	Physician’s prior experience	165 (31.3)	Perceived clinical benefit	232 (36.3)
Concerns about drug cost	121 (12.4)	Concerns about drug cost	108 (12.5)	Perceived clinical benefit	134 (25.4)	Physician’s prior experience	112 (17.5)
Case 7							
No antivirals				Antivirals			
Pre (n = 893)		Post (n = 658)		Pre (n = 607)		Post (n = 842)	
Mild symptoms	813 (91.0)	Mild symptoms	580 (88.1)	Perceived clinical benefit	249 (41.0)	Perceived clinical benefit	523 (62.1)
Concerns about drug cost	130 (14.6)	Concerns about drug cost	101 (15.3)	Physician’s prior experience	191 (31.5)	Physician’s prior experience	147 (17.5)
No perceived clinical benefit	43 (4.8)	No perceived clinical benefit	31 (4.7)	Concerns about drug cost	134 (22.1)	Concerns about drug cost	115 (13.7)
Case 8							
No antivirals				Antivirals			
Pre (n = 945)		Post (n = 667)		Pre (n = 555)		Post (n = 833)	
Mild symptoms	827 (87.5)	Mild symptoms	554 (83.1)	Concerns regarding drug interactions	187 (33.7)	Concerns regarding drug interactions	376 (45.1)
Concerns about drug cost	122 (12.9)	Concerns about drug cost	100 (15.0)	Physician’s prior experience	168 (30.3)	Perceived clinical benefit	281 (33.7)
Concerns regarding drug interactions	88 (9.3)	Concerns regarding drug interactions	82 (12.3)	Perceived clinical benefit	144 (25.9)	Physician’s prior experience	126 (15.1)

The data are presented as numbers (%). Pre, before viewing the educational film; post, after viewing the educational film.

**Table 4:** Reasons for prescribing or withholding antivirals among patients with hypertension and hyperlipidemia before and after viewing educational film.

Case 9							
No Antivirals				Antivirals			
Pre (n = 1033)		Post (n = 989)		Pre (n = 467)		Post (n = 511)	
Mild symptoms	934 (90.4)	Mild symptoms	834 (84.3)	Perceived clinical benefit	173 (37.0)	Perceived clinical benefit	250 (48.9)
Vaccination within the past year	178 (17.2)	Vaccination within the past year	324 (32.8)	Physician’s prior experience	166 (35.5)	Physician’s prior experience	108 (21.1)
Concerns about drug cost	146 (14.1)	Concerns about drug cost	121 (12.2)	Concerns about drug cost	112 (24.0)	Concerns about drug cost	92 (18.0)
Case 10							
No antivirals				Antivirals			
Pre (n = 1070)		Post (n = 1021)		Pre (n = 430)		Post (n = 479)	
Mild symptoms	935 (87.4)	Mild symptoms	848 (83.1)	Concerns regarding drug interactions	144 (33.5)	Concerns regarding drug interactions	176 (36.7)
Vaccination within the past year	231 (21.6)	Vaccination within the past year	380 (37.2)	Physician’s prior experience	140 (32.6)	Perceived clinical benefit	151 (31.5)
Concerns about drug cost	151 (14.1)	Concerns about drug cost	120 (11.8)	Perceived clinical benefit	113 (26.3)	Physician’s prior experience	101 (21.1)
Case 11							
No antivirals				Antivirals			
Pre (n = 986)		Post (n = 824)		Pre (n = 514)		Post (n = 676)	
Mild symptoms	898 (91.1)	Mild symptoms	747 (90.7)	Perceived clinical benefit	202 (39.3)	Perceived clinical benefit	367 (54.3)
Concerns about drug cost	147 (14.9)	Concerns about drug cost	122 (14.8)	Physician’s prior experience	160 (31.1)	Physician’s prior experience	144 (21.3)
No perceived clinical benefit	38 (3.9)	No perceived clinical benefit	46 (5.6)	Concerns about drug cost	119 (23.2)	Concerns about drug cost	111 (16.4)
Case 12							
No antivirals				Antivirals			
Pre (n = 1017)		Post (n = 851)		Pre (n = 483)		Post (n = 649)	
Mild symptoms	903 (88.8)	Mild symptoms	739 (86.8)	Concerns regarding drug interactions	185 (38.3)	Concerns regarding drug interactions	280 (43.1)
Concerns about drug cost	144 (14.2)	Concerns about drug cost	124 (14.6)	Physician’s prior experience	142 (29.4)	Perceived clinical benefit	208 (32.0)
Concerns regarding drug interactions	112 (11.0)	Concerns regarding drug interactions	105 (12.3)	Perceived clinical benefit	117 (24.2)	Physician’s prior experience	133 (20.5)

The data are presented as numbers (%). Pre, before viewing the educational film; post, after viewing educational film.

**Table 5:** Reasons for prescribing or withholding antivirals among patients without risk factors for disease progression before and after viewing educational film.

Case 13							
No Antivirals				Antivirals			
Pre (n = 1193)		Post (n = 1178)		Pre (n = 307)		Post (n = 322)	
Mild symptoms	1069 (89.6)	Mild symptoms	1018 (86.4)	Physician’s prior experience	103 (33.6)	Perceived clinical benefit	124 (38.5)
Absence of risk factors for severe disease	282 (23.6)	Absence of risk factors for severe disease	342 (29.0)	Perceived clinical benefit	94 (30.6)	Physician’s prior experience	90 (28.0)
Vaccination within the past year	212 (17.8)	Vaccination within the past year	296 (25.1)	Concerns about drug cost	78 (25.4)	Concerns about drug cost	72 (22.4)
Case 14							
No antivirals				Antivirals			
Pre (n = 1220)		Post (n =1194)		Pre (n = 280)		Post (n = 306)	
Mild symptoms	1050 (86.1)	Mild symptoms	1000 (83.8)	Physician’s prior experience	90 (32.1)	Perceived clinical benefit	105 (34.3)
Absence of risk factors for severe disease	380 (31.1)	Absence of risk factors for severe disease	412 (34.5)	Perceived clinical benefit	80 (28.6)	Concerns regarding drug interactions	84 (27.5)
Vaccination within the past year	232 (19.0)	Vaccination within the past year	356 (29.8)	Concerns regarding drug interactions	71 (25.4)	Physician’s prior experience	73 (23.9)
Case 15							
No antivirals				Antivirals			
Pre (n = 1136)		Post (n = 1030)		Pre (n = 364)		Post (n = 470)	
Mild symptoms	1022 (90.0)	Mild symptoms	914 (88.7)	Physician’s prior experience	128 (35.2)	Perceived clinical benefit	212 (45.1)
Absence of risk factors for severe disease	335 (29.5)	Absence of risk factors for severe disease	327 (31.7)	Perceived clinical benefit	114 (31.3)	Physician’s prior experience	114 (24.3)
Concerns about drug cost	152 (13.4)	Concerns about drug cost	149 (14.5)	Concerns about drug cost	102 (28.0)	Concerns about drug cost	110 (23.4)
Case 16							
No antivirals				Antivirals			
Pre (n = 1164)		Post (n = 1065)		Pre (n = 336)		Post (n = 435)	
Mild symptoms	1018 (87.5)	Mild symptoms	930 (87.3)	Concerns regarding drug interactions	111 (33.0)	Perceived clinical benefit	146 (33.6)
Absence of risk factors for severe disease	334 (28.7)	Absence of risk factors for severe disease	350 (32.9)	Physician’s prior experience	107 (31.8)	Concerns regarding drug interactions	143 (32.9)
Concerns about drug cost	152 (13.1)	Concerns about drug cost	149 (14.0)	Perceived clinical benefit	89 (26.5)	Physician’s prior experience	90 (20.7)

The data are presented as numbers (%). Pre, before viewing the educational film; post, after viewing the educational film.

**Table 6:** The details of the 16 fictitious scenarios in the film featuring adult patients with COVID-19 with varying risk factors for severe disease and vaccination statuses.

Case No.	Risk Factors for Severe Disease Progression	Vaccination Within the Past Year	Concomitant Use of CYP3A-Metabolized Drug
1	Immunosuppressed status	Yes	No
2			Yes
3		No	No
4			Yes
5	Obesity	Yes	No
6			Yes
7		No	No
8			Yes
9	Hypertension/hyperlipidemia	Yes	No
10			Yes
11		No	No
12			Yes
13	None	Yes	No
14			Yes
15		No	No
16			Yes

**Table 7:** Summary of antiviral drugs investigated in hypothetical scenario.

Drug	Indication	Mechanism of Action	Dose	Duration	Timing of Administration	Interactions with CYP3A-Metabolized Drugs
Nirmatrelvir/ritonavir	Mild to moderate COVID-19 with risk factors for severe disease	3C-like protease inhibitor	Nirmatrelvir 300 mg plus ritonavir 100 mg, twice a day	5 days	Within 5 days of symptom onset	Yes
Molnupiravir	Mild to moderate COVID-19 with risk factors for severe disease	RNA-dependent RNA polymerase inhibitor	800 mg every 12 h, twice a day	5 days	Within 5 days of symptom onset	No
Ensitrelvir	Mild COVID-19	3C-like protease inhibitor	375 mg on day 1, then 125 mg per day	5 days	Within 3 days of symptom onset	Yes

## Data Availability

The datasets used and analyzed during the current study are available from the corresponding author upon reasonable request.

## Supplementary Materials

The following supporting information can be downloaded at: https://www.mdpi.com/article/10.3390/antibiotics14030276/s1, Figure S1: Rates of antiviral prescription by generalists among patients with an immunosuppressed status before and after viewing the educational film; Figure S2: Rates of antiviral prescription by generalists among patients with obesity before and after viewing the educational film; Figure S3: Rates of antiviral prescription by generalists among patients with hypertension or hyperlipidemia before and after viewing the educational film; Figure S4: Rates of antiviral prescription by generalists among patients without risk factors for disease progression before and after viewing the educational film; Figure S5: Rates of antiviral prescription by nongeneralists among patients with an immunosuppressed status before and after viewing the educational film; Figure S6: Rates of antiviral prescription by nongeneralists among patients with obesity before and after viewing the educational film; Figure S7: Rates of antiviral prescription by nongeneralists among patients with hypertension or hyperlipidemia before and after viewing the educational film; Figure S8: Rates of antiviral prescription by nongeneralists among patients without risk factors for disease progression before and after viewing the educational film.

## Abbreviations

The following abbreviations are used in this manuscript:

SARS-CoV-2	Severe acute respiratory syndrome coronavirus 2
COVID	Coronavirus disease 2019
JRS	Japanese Respiratory Society
RCT	Randomized controlled trial
