# Supplementary material for: Influence of Educational Films on Antiviral Prescription for COVID-19: Insights from Web-Based Survey in Japan
Source: Antibiotics (Basel). 2025 Mar 7;14(3):276. doi: 10.3390/antibiotics14030276 (PMC11939197; doi:10.3390/antibiotics14030276)
Supplement: Supplementary file 1 [file antibiotics-14-00276-s001.zip › antibiotics-3502426-supplementary.pptx]

## Slide 1
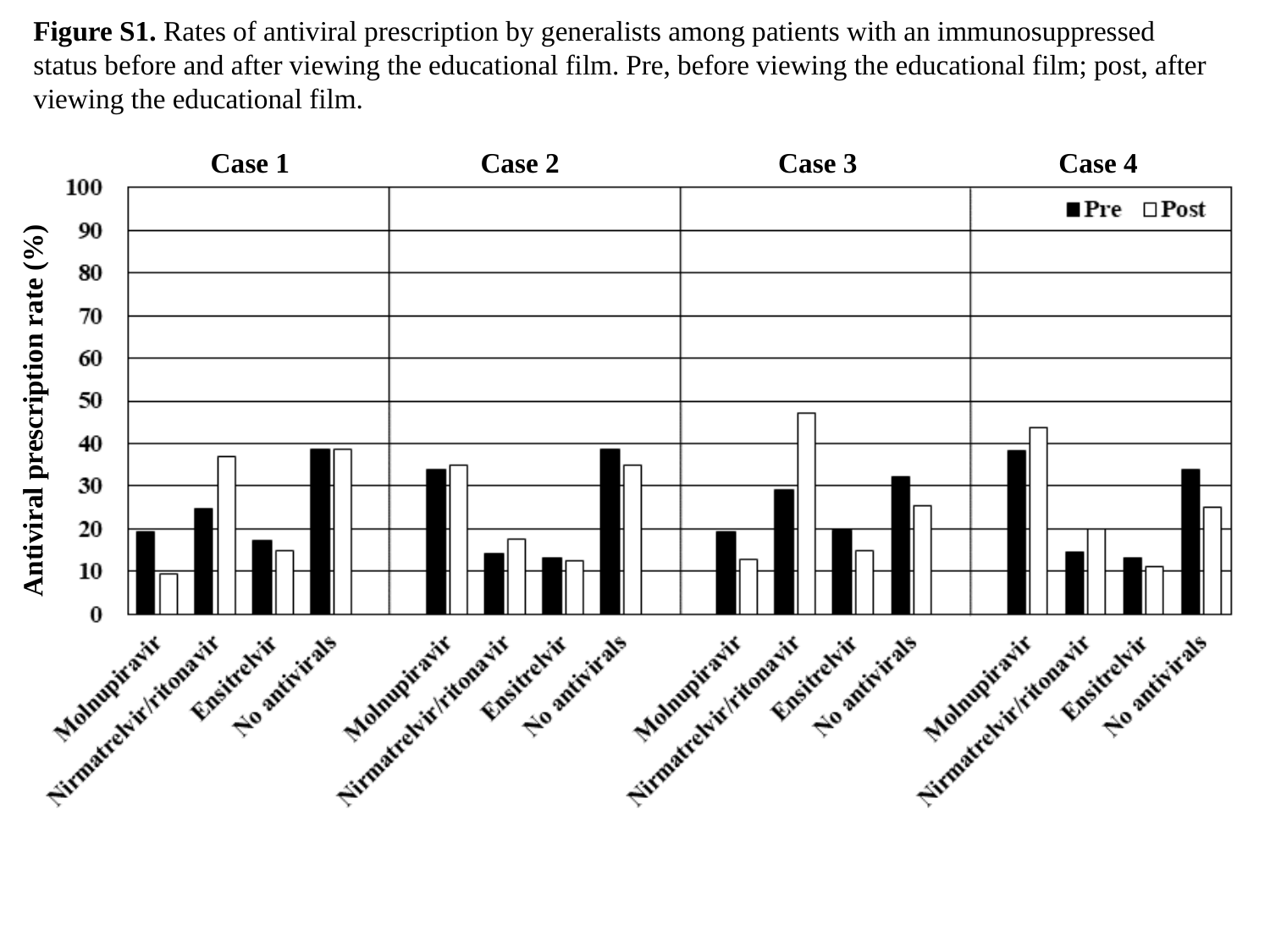

Figure S1. Rates of antiviral prescription by generalists among patients with an immunosuppressed status before and after viewing the educational film. Pre, before viewing the educational film; post, after viewing the educational film.
Case 1
Case 3
Case 4
Case 2
Antiviral prescription rate (%)

## Slide 2
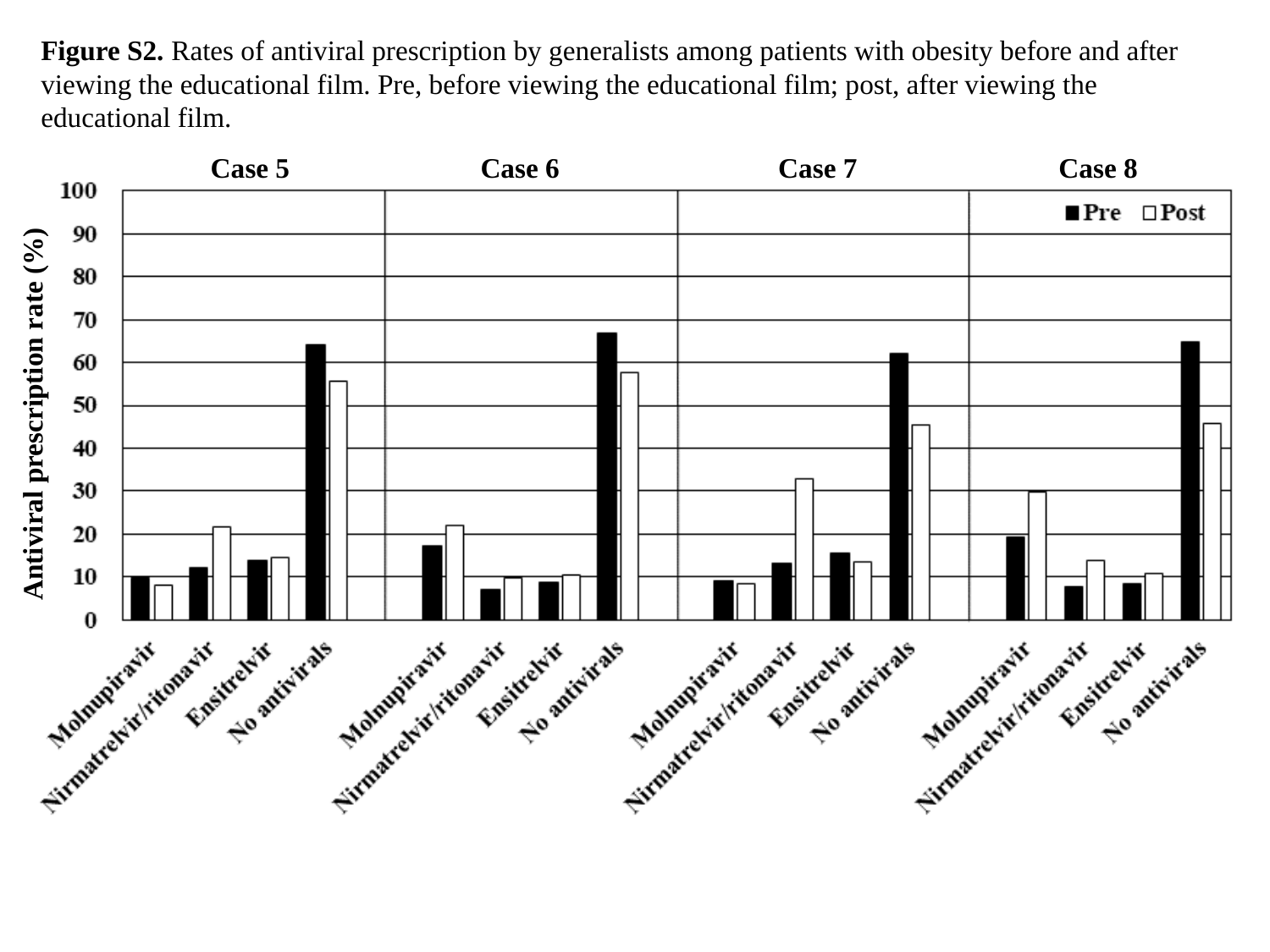

Figure S2. Rates of antiviral prescription by generalists among patients with obesity before and after viewing the educational film. Pre, before viewing the educational film; post, after viewing the educational film.
Case 8
Case 5
Case 7
Case 6
Antiviral prescription rate (%)

## Slide 3
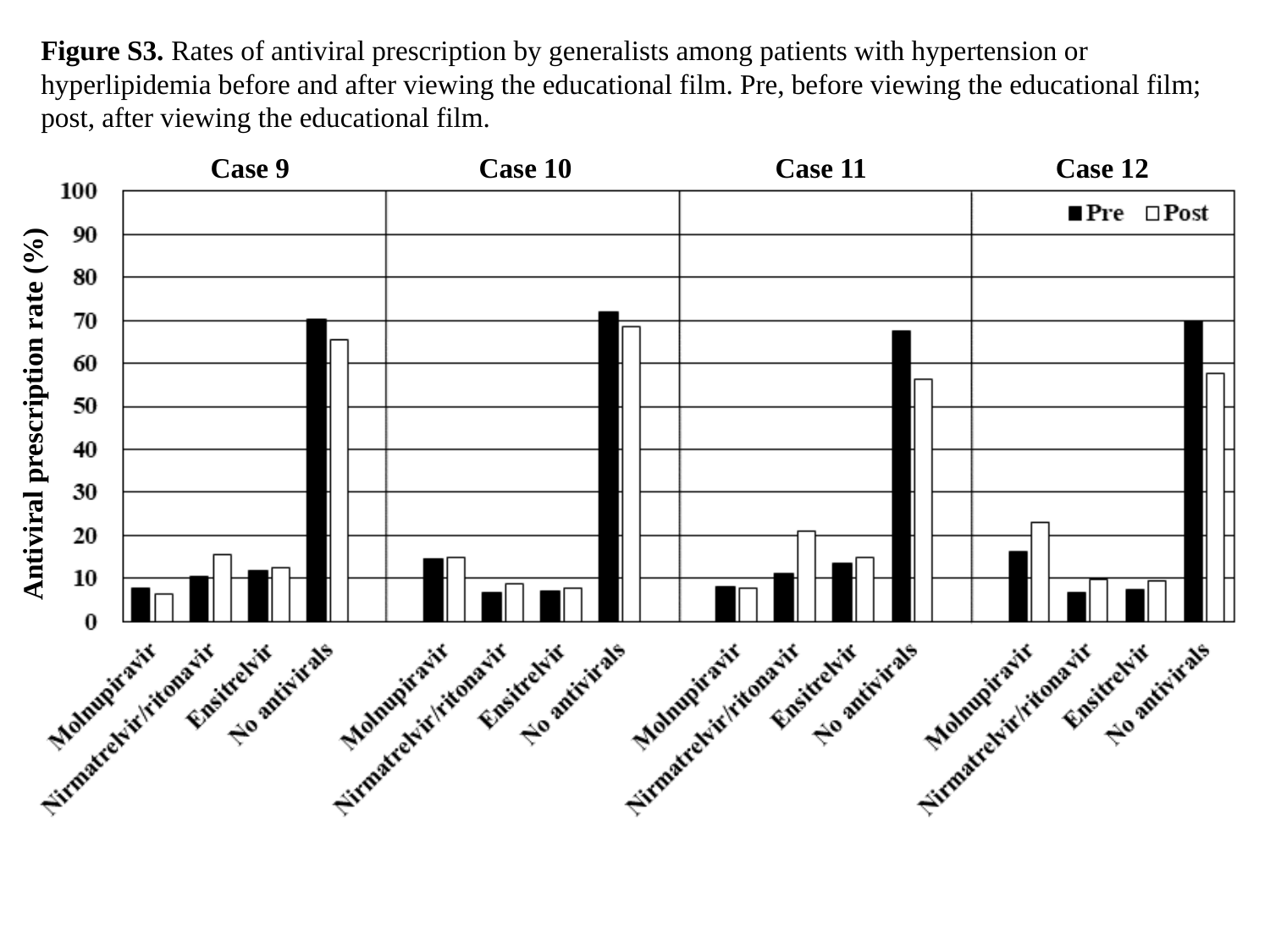

Figure S3. Rates of antiviral prescription by generalists among patients with hypertension or hyperlipidemia before and after viewing the educational film. Pre, before viewing the educational film; post, after viewing the educational film.
Case 12
Case 9
Case 11
Case 10
Antiviral prescription rate (%)

## Slide 4
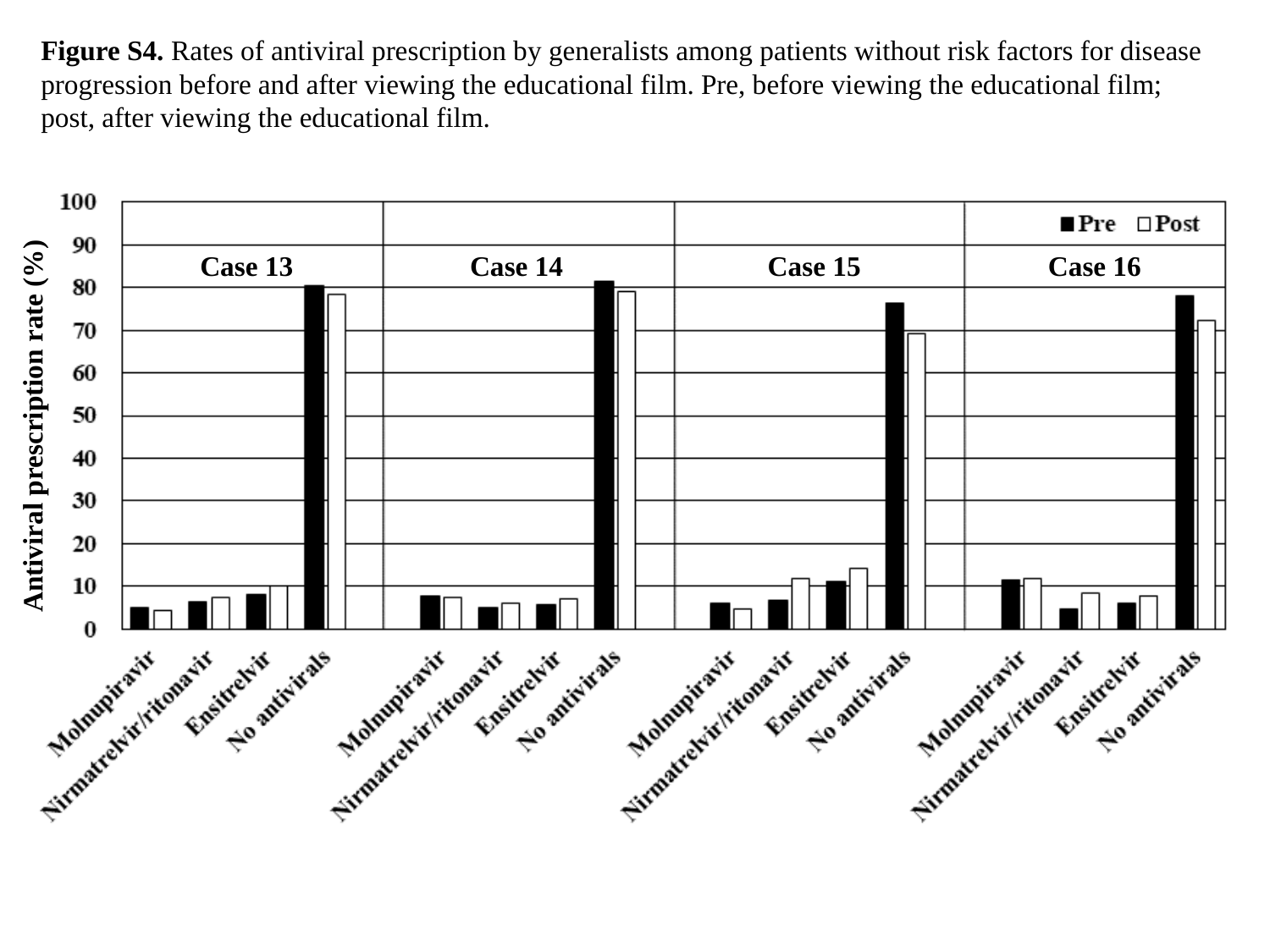

Figure S4. Rates of antiviral prescription by generalists among patients without risk factors for disease progression before and after viewing the educational film. Pre, before viewing the educational film; post, after viewing the educational film.
Case 13
Case 14
Case 15
Case 16
Antiviral prescription rate (%)

## Slide 5
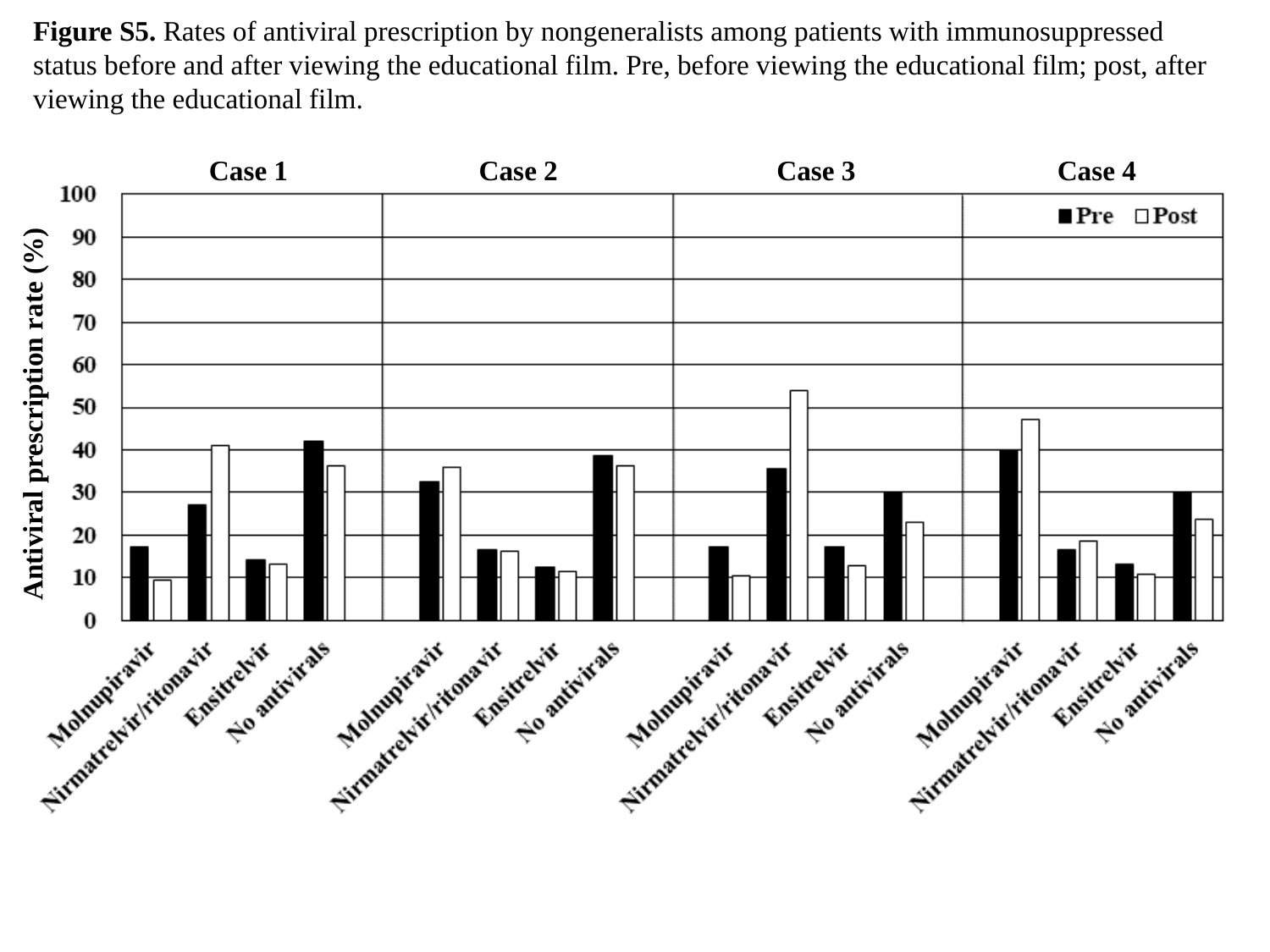

Figure S5. Rates of antiviral prescription by nongeneralists among patients with immunosuppressed status before and after viewing the educational film. Pre, before viewing the educational film; post, after viewing the educational film.
Case 4
Case 3
Case 1
Case 2
Antiviral prescription rate (%)

## Slide 6
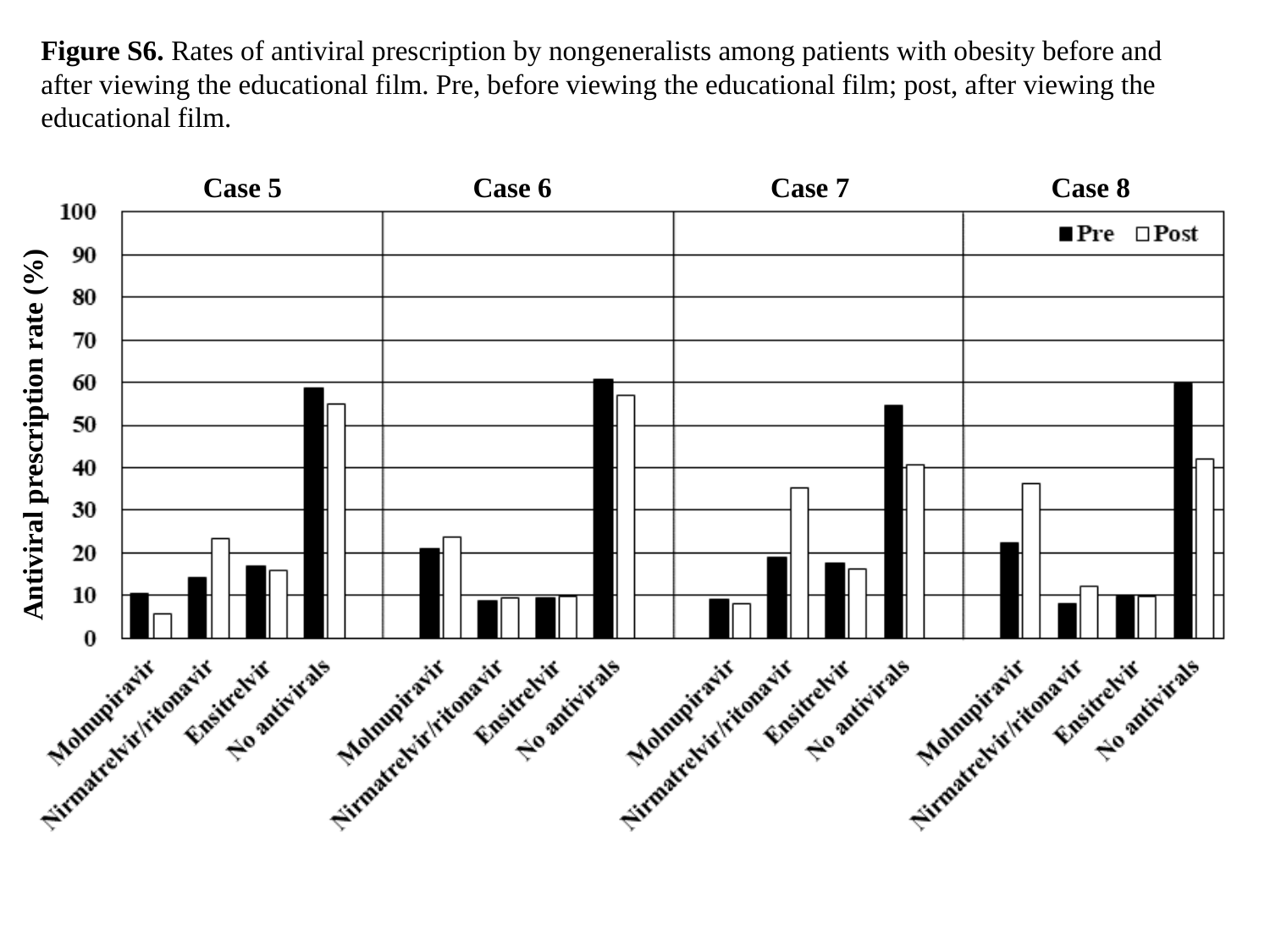

Figure S6. Rates of antiviral prescription by nongeneralists among patients with obesity before and after viewing the educational film. Pre, before viewing the educational film; post, after viewing the educational film.
Case 7
Case 5
Case 8
Case 6
Antiviral prescription rate (%)

## Slide 7
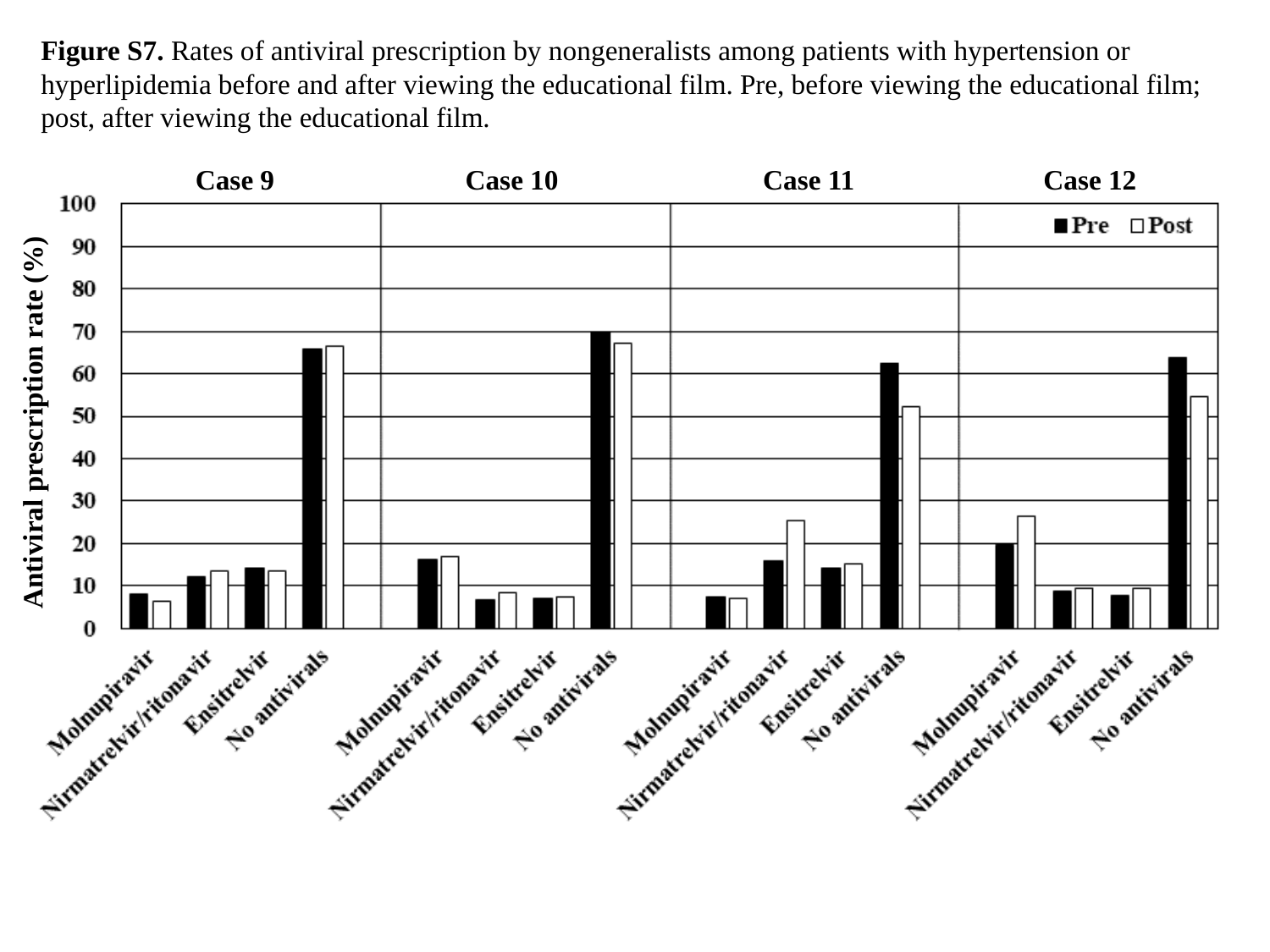

Figure S7. Rates of antiviral prescription by nongeneralists among patients with hypertension or hyperlipidemia before and after viewing the educational film. Pre, before viewing the educational film; post, after viewing the educational film.
Case 12
Case 9
Case 10
Case 11
Antiviral prescription rate (%)

## Slide 8
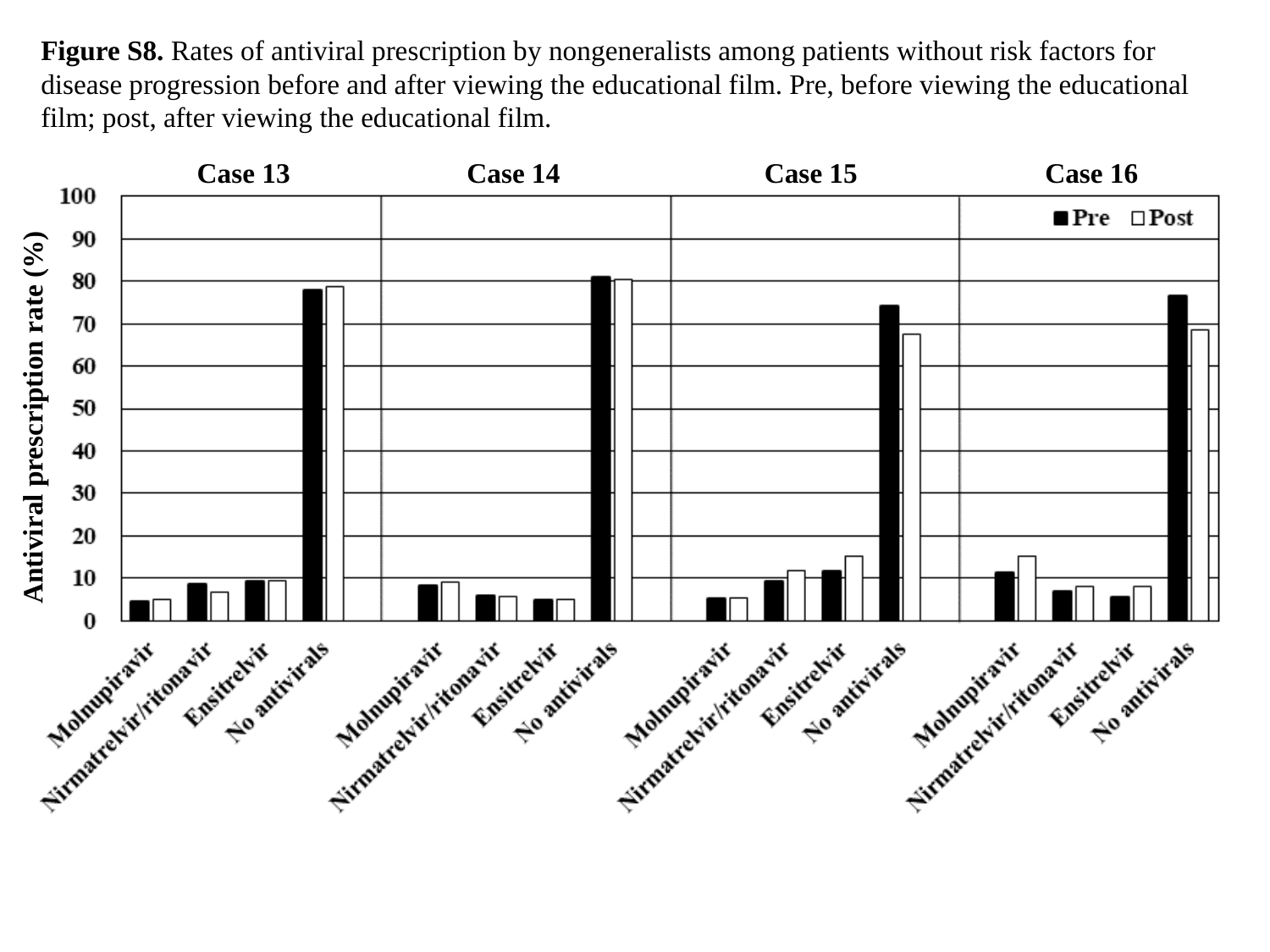

Figure S8. Rates of antiviral prescription by nongeneralists among patients without risk factors for disease progression before and after viewing the educational film. Pre, before viewing the educational film; post, after viewing the educational film.
Case 15
Case 16
Case 13
Case 14
Antiviral prescription rate (%)
